# Microglia-Derived Exosomes Improve Spinal Cord Functional Recovery after Injury via Inhibiting Oxidative Stress and Promoting the Survival and Function of Endothelia Cells

**DOI:** 10.1155/2021/1695087

**Published:** 2021-08-25

**Authors:** Wei Peng, Liyang Wan, Zixiang Luo, Yong Xie, Yudong Liu, Tingmo Huang, Hongbin Lu, Jianzhong Hu

**Affiliations:** ^1^Department of Spine Surgery and Orthopaedics, Xiangya Hospital, Central South University, Changsha, China; ^2^Key Laboratory of Organ Injury, Aging and Regenerative Medicine of Hunan Province, 410008 Changsha, China; ^3^Hunan Engineering Research Center of Sports and Health, China; ^4^National Clinical Research Center for Geriatric Disorders, Xiangya Hospital, Central South University, Changsha, China; ^5^Department of Sports Medicine, Xiangya Hospital, Central South University, Changsha 410008, China

## Abstract

Traumatic spinal cord injury (SCI) is a devastating disease of the central nervous system with long-term disability and high mortality worldwide. Revascularization following SCI provides nutritional supports to rebuild and maintain the homeostasis of neuronal networks, and the subsequent promotion of angiogenesis is beneficial for functional recovery. Oxidative stress drastically produced following SCI has been contributed to endothelial dysfunction and the limited endogenous repair of microvasculature. Recently, exosomes, being regarded as potential therapeutic candidates for many kinds of diseases, have attracted great attentions due to its high bioavailability, safety, and stability. Microglia have been reported to exhibit proangiogenic function and guide the forming of vasculature during tissue repair. However, the specific role of microglia-derived exosomes (MG-Exos) played in SCI is still largely unknown. In the present study, we aimed to evaluate whether MG-Exos could protect spinal cord microvascular endothelial cells (SCMECs) against the toxic effects of oxidative stress, thus promote SCMECs' survival and function. We also investigated the protective effects of MG-Exos in the mouse model of SCI to verify their capability. Our results demonstrated that MG-Exo treatment significantly decreased the level of oxidative stress (ROS), as well as did the protein levels of NOX2 when bEnd.3 cells were exposed to H_2_O_2_-induced oxidative stress *in vitro* and *in vivo*. Functional assays showed that MG-Exos could improve the survival and the ability of tube formation and migration in H_2_O_2_-induced bEnd.3 *in vitro*. Moreover, MG-Exos exhibited the positive effects on vascular regeneration and cell proliferation, as well as functional recovery, in the mouse model of SCI. Mechanically, the keap1/Nrf2/HO-1 signaling pathway was also investigated in order to unveil its molecular mechanism, and the results showed that MG-Exos could increase the protein levels of Nrf2 and HO-1 via inhibiting the keap1; they also triggered the expression of its downstream antioxidative-related genes, such as *NQo1*, *Gclc*, *Cat*, and *Gsx1*. Our findings indicated that MG-Exos exerted an antioxidant effect and positively modulated vascular regeneration and neurological functional recovery post-SCI by activating keap1/Nrf2/HO-1 signaling.

## 1. Introduction

Traumatic spinal cord injury (SCI) is a devastating disease of the central nervous system, which may cause major motor, sensory and autonomic dysfunctions, and bring far-reaching adverse consequences for patients, families, and society [1]. Epidemiological data showed that the number of prevalent cases of SCI, which had been rising for years, was approximately 27.04 million, and there were estimatedly 0.93 million new cases annually throughout the world [2].

Contusion injury, followed by hypoxia, ischemia-reperfusion injury (SCIRI), and microenvironment imbalance, can cause the production and release of large amounts of free oxygen radicals and reactive oxygen species (ROS) [3, 4]. These strong oxidants accumulate in the tissues leading to topical microvasculature disorder and thus the dysfunction of myelin sheaths of nerve cells [5–8]. Studies have shown that oxidative stress is the main pathophysiological events leading to secondary damage for neural tissue and the parenchyma of the spinal cord in the injury epicenter [9, 10].

Posttraumatic vascularization ensures tremendous trophic support to build up and maintain homeostasis of neuronal networks [11–13]. Regenerating axons have been shown to grow along blood vessels [12, 14, 15]. However, high level of oxidative stress and poor microenvironment limited the endogenous repair of microvasculature and the regeneration of axons following SCI [16, 17]. Thus, quickly balancing the oxidative stress of the spinal cord is an important way to improve angiogenesis, restore blood flow, and protect the function of spinal cord microvasculature and nerve cells.

Recently, extracellular vesicles (EVs), such as exosomes and small microvesicles, are recognized as novel tools of intercellular communication [18]. Depending on their parental cells, these EVs contain a variety of specific sets of substances, including proteins, lipids, metabolites, and nucleic acids [18, 19]. Previous evidences showed that exosomes derived from mesenchymal stem cells (MSCs) or endothelial progenitor cells (EPCs) or adipose-derived stem cells (ADSCs) can protect cells such as epidermal keratinocytes and endothelial cells against the toxic effects of high oxidative stress and promote their survival rates [20–22]. In central nervous system, the neighbouring spatial localization of microglia and microvascular endothelial cells suggested an inseparable role of microglia in vascular angiogenesis and vasculopathy [23]. Mounting evidences are suggesting that microglia exhibit the proangiogenic function through forming perivascular clusters and secreting multiple factors such as VEGF (vascular endothelial growth factor), basigin-2, and FGF2 (fibroblast growth factor 2), which makes them key regulators and guides of angiogenesis in ischemic stroke and traumatic brain injury (TBI) [23–27]. Deletion of microglia in mice was found to have reduced vascular branch points in retina [28]. In the recent, Xu et al. have reported that microglia-derived exosomes (MG-Exos) can reduce the avascular areas of retinal in retinopathy of prematurity [29]. However, proangiogenic properties of MG-Exo in SCI angiogenesis and its underlying mechanism have not yet been investigated.

In this study, we determined that MG-Exos can protect the endothelial cells of spinal cord microvascular from the toxic effects of high oxidative stress and promote the function along with the survival rate of the endothelial cells.

## 2. Methods

### 2.1. Isolation, Culture, and Identification of Microglia

The primary mouse microglia were isolated and maintained as Saura et al. described [30]. Briefly, ten 1-day-old Bl/C57 mice were sacrificed by cervical dislocation. Afterwards, the mice's brains were isolated, the meninges and vessels were carefully separated with micro tweezers under microscope. The tissue was minced with a 1 ml tip pipette until the media became milky. Then, the tissue was digested with 0.25% trypsin (Sigma-Aldrich, USA) for 15 min at 37°C. After centrifuged and discarded supernatant, cells were seeded to cell culture plates with Dulbecco's Modified Eagle's Medium-F12 nutrient mixture (DMEM-F12, Gibco, USA) containing 10% fetal bovine serum (FBS; Gibco), sodium pyruvate (1×), and 0.08% (w/v) gentamicin sulfate (Sigma). After cultured 10–12 days, cells were digested with 0.25% trypsin diluted 1 : 4 in DMEM-F12 for 25 min at 37°C to purify microglia.

The expression of surface marker proteins (including F4/80 (Abcam, USA) and Iba-1 (Wako, Japan)) on microglia was detected by immunofluorescence. The expression of surface marker proteins (including CD45, F4/80, CD11b, and CD68) on microglia was detected by flow cytometry.

### 2.2. Culture of bEnd.3 Cell Line

The bEnd.3 cells (brain-derived endothelial cells.3), immortalized mouse brain endothelial cells, were obtained from Advance Research Centre of Central South University (Changsha, China). They were grown in Dulbecco's Modified Eagle's Medium (DMEM) supplemented with 10% FBS, 1% penicillin, and streptomycin (Gibco). The bEnd.3 cells were maintained in a humidified incubator at 37°C with 5% CO_2_ and 95% air.

### 2.3. Isolation and Identification of Microglia Exosomes

Cells were incubated in the completed medium containing exosome-depleted FBS (Vivacell, China) for 48 h. Microglia exosomes were collected from the completed medium by differential centrifugation as described previously [31]. In brief, the completed medium was centrifuged at 800×g for 10 min and 3000×g for 30 min at 4°C. After centrifugation at 10,000×g for 1 h at 4°C, the supernatant was filtered using a 0.22 *μ*m filter (Millipore, USA) to remove cells and debris. The exosomes were purified twice by centrifugation at 140,000×g for 3 h and then resuspended for further characterization.

A transmission electron microscope (TEM; FEI company, USA) was further used to identify the form of the exosomes. Nanoparticle tracking analysis (NTA; ZetaView, Germany) was used to measure exosomes' diameter and particle number. The exosome markers CD9, tumor susceptibility gene 101 (TSG101), and CD63 were detected by western blotting analysis.

### 2.4. Exosome Uptake Assay

MG-Exos were labeled with a red fluorescent lipophilic dye PKH67 (Sigma) to monitor the motion of the exosomes. MG-Exos were resuspended in sterile PBS and incubated with 5 *μ*M PKH67 for 15 min. Then, the suspension containing labeled exosomes was washed twice at 140,000×g for 90 min and resuspended in sterile PBS and used for the uptake experiment.

The bEnd.3 cells were seeded on chambered culture slides in 24-well cell culture plates until reaching 80% confluence and then incubated in the completed medium containing 200 *μ*g/ml PKH67-labeled exosomes at 37°C for 12 h. Culture slides were fixed with 4% paraformaldehyde (PFA; Biosharp, China) for 20 min and then incubated with 5% BSA (Biofroxx, Germany) in PBS for 30 min to block nonspecific staining. Nuclei were stained with DAPI (GeneTex Inc., USA), and signals were analyzed with a fluorescence microscope (Zeiss Apotome 2, Zeiss, Germany).

### 2.5. CCK-8 Assay

100 *μ*l cell suspension (5 × 10^3^ cells) was seeded into each well of 96-well culture plates and treated with exosomes (200 *μ*g/ml) or PBS. Four wells of replicates were set up in each group, and a group without cells served as the blank. After incubated in completed medium with 500 *μ*M H_2_O_2_ for 2, 4, 6, 12, and 24 hours, 10 *μ*l cell counting kit-8 reagent (CCK-8; 7Sea Biotech, China) was added to the culture medium of each well. After incubation at 37°C for 2 h, the absorbance was detected at 450 nm by a microplate reader (Thermo Fisher Scientific, USA) and cell's survival rates were represented through the mean absorbance value of each well minus the blank value.

### 2.6. Transwell Chamber Migration Assay

Six-well transwell chamber with 8 mm filter inserts (Corning, China) was used to observe bEnd.3 migration. Cells were digested into suspensions with trypsin; the upper chambers were filled with 2 × 10^5^ cells/ml containing DMEM (Gibco) and 1% FBS (Gibco), and the lower chambers were filled with 600 *μ*l medium containing 10% FBS (Gibco) and MG-Exos (100 *μ*g/ml) or Vehicle. After culturing the cells in completed medium with 500 *μ*M H_2_O_2_ for 24 h, the nonmigrated cells on the upper chambers were removed, and the migrated cells on the lower surface were fixed in 4% PFA for 20 min. And then, the migrated cells were stained with 0.1% crystal violet for 20 min and quantified by counting six random fields.

### 2.7. Scratch Wound Migration Assay

bEnd.3 cells were seeded on six-well plates until confluence reaching at 90%. The monolayer cells were scratched in a cross shape using a P-200 tip. After cultured in completed medium with 500 *μ*M H_2_O_2_ for 0 h, 6 h, and 12 h after scratching, the wound width was recorded with Microscope (Primovert, Zeiss, Germany). The ImageJ (National Institutes of Health) was used for measuring the distance between the sides of the scratch in six random fields.

### 2.8. Tube Formation Assay

50 *μ*l precooled growth factor-reduced Matrigel (BD) was added to a 96-well plate. Then, bEnd.3 (2 × 10^4^ cells/well) were seeded onto the Matrigel and treated with MG-Exos or Vehicle. After cultured in the medium with 500 *μ*M H_2_O_2_ for 12 h, the network formation was assessed using a bright-field microscope (Zeiss). The ability to form capillary-like structures was quantified by determining the number of branch points, tubule lengths, and loops in six randomly chosen microscopic fields using the ImageJ software.

### 2.9. Measurement of Intracellular ROS Levels

The level of intracellular reactive oxygen species (ROS) was measured using Reactive Oxygen Species Assay Kit (DCFH-DA) (Solarbio, China), in which the intensity of fluorescence is proportional to ROS level. bEnd.3 were first seeded on 24-well plates. When they reached 70 to 80% confluence, they were cultured with the completed medium with 500 *μ*M H_2_O_2_ and MG-Exos or Vehicle for 24 h. Then, DCFH-DA was added and incubated for 30 min in dark at 37°C, followed by a fixation with 4% PFA for 20 min. After washing with PBS, cells were stained with DAPI. Fluorescence was measured using a fluorescence microscope (Zeiss Apotome 2).

In vivo, the spinal cords of contusion or sham were collected and digested into the supernatant by using the Collagenase/Dispase (Sigma). After red blood cell lysis and cells counts, DCFH-DA was added and incubated for 30 min in dark at 37°C. After washing three times with the medium, the cells were resuspended in PBS. Fluorescence was detected at 525 nm by a microplate reader (Thermo Fisher Scientific).

### 2.10. Animals and Surgical Procedures

All animal protocols were approved by the Ethics Committee of Central South University (CSU) for Scientific Research. Experiments were conducted using 8-12-week-old female C57BL/6 (Charles River) mice. An SCI model of female mice was established, as previously described [32]. In brief, female mice were deeply anesthetized with ketamine/xylazine/acepromazine (50 : 5 : 1 mg/kg) by intraperitoneal (i.p.) injection. After a laminectomy used to expose the spinal cord at the 10th thoracic vertebrae (T10), moderate contusion injuries of the spinal cord were created using a modified Allen's weight drop apparatus (10 g weight at a vertical height of 20 mm, 10 g × 20 mm). Mice in the sham group were subjected to laminectomy without contusion. Bladders of animal were manually voided twice per day until full voluntary or autonomic voiding was obtained, and antibiotic (penicillin sodium; North China Pharmaceutical, China) was administered daily for 3 days postsurgery.

Mice were randomly and blindly divided into two groups (*n* = 8/group for each time point) and treated with 200 *μ*l of Vehicle or 200 *μ*g of MG-Exos in 200 *μ*l, administered postsurgery by tail vein injection.

The functional recovery from SCI was measured at different time points through locomotor behavioral assessments, electrophysiological test, and H&E, as well as immunofluorescence analyses.

### 2.11. Evaluation of the Locomotive Function

To evaluate the locomotive function of hindlimbs, BMS (Basso Mouse Scale) system was used before surgery and 1, 3, 7, 14, 21 and 28 days post-SCI [33]. The BMS ranges from 0 (complete paralysis) to 9 (normal locomotion) points. The 11-point BMS subscore which included frequency of plantar stepping, coordination, paw position, trunk stability, and tail position was also assessed and supplemented the main scale. Each mouse was observed 5 min, and the average BMS scores and subscores were recorded by two well-trained investigators who were familiar with the BMS scores and blinded to the experimental design.

### 2.12. Neuroelectrophysiology

To evaluate movement recovery after SCI, the MEPs (motor evoked potentials) of hindlimbs were recorded by electromyography conducted at 4 weeks post-SCI as described in our previous research [34]. Briefly, after anesthesia, the stimulating electrodes were secured onto the surface of the skull corresponding to the motor area of the cerebral cortex, while the recording electrodes were inserted into the tibialis anterior muscle of the contralateral hindlimb. The reference electrode was placed into subcutaneous tissue between the stimulating and recording electrodes. The mean MEP values (including amplitude and latency period) were captured before surgery and 4 weeks postsurgery.

### 2.13. Quantitative Real-Time PCR (qRT-PCR) Analysis

Total RNA was extracted using TRIzol Reagent (Invitrogen, USA). Single-strand cDNA was reverse transcribed from 1 *μ*g total RNA from each sample using GoScript™ Reverse Transcription System (Promega Corporation, USA) according to the manufacturer's instructions. qRT-PCR was performed by GoScript™ qPCR Master Mix (Promega) using a BRYT Green master mix. All reactions were processed and analyzed on a real-time PCR System (FTC-3000, Funglyn Biotech Inc., Canada). The expression level of target genes was normalized to GAPDH, and relative gene expression was calculated using the 2^–*ΔΔ*CT^ method. Primer sequences used for qRT-PCR were shown in Table 1.

### 2.14. Western Blotting

Cells and exosomes were lysed by RIPA buffer containing protease and phosphatase inhibitor (Solarbio). After centrifugation at 10,000×g for 10 min, the supernatant was collected, and the protein concentration of which was detected using the BCA Protein Assay Kit (Solarbio). Then, the supernatant was diluted with protein loading buffer (5×; Beyotime Biotechnology, China) and heated at 100°C for 10 min. Proteins were separated by SDS-PAGE gels 10~12%, transferred to polyvinylidene fluoride membrane (Millipore). The membranes were blocked with 5% milk in TBST (Tris-buffered saline, 10 mM Tris-HCl pH 7.5, 150 mM NaCl, and 0.1% Tween-20) for 60 min and then incubated with primary antibodies at 4°C overnight. Primary antibodies and dilutions were used as follows: rabbit anti-NOX2 (1 : 1,000), rabbit anti-Nrf2 (1 : 1,000), rabbit anti-keap1 (1 : 2,000), rabbit anti-HO-1 (1 : 1,000), rabbit anti-TSG101 (1 : 2,000), rabbit anti-CD63 (1 : 1,000), and rabbit anti-CD9 (1 : 1,000). Blots were washed with TBST and incubated with peroxidase-conjugated goat anti-rabbit IgG (1 : 5,000). Equal loading of proteins was assessed using a rabbit anti-*β*-actin (1 : 5,000). All primary antibodies purchased from Proteintech (China). The immunoreactive bands were visualized by using the Enhanced Chemiluminescence Reagent (Millipore) and imaged by ChemiDoc XRS Plus luminescent image analyzer (Bio-Rad, England).

### 2.15. Flow Cytometry

Microglia were first blocked Fc receptors with anti-mouse CD16/CD32 antibody (BD, USA) for 15 min at 4°C in FACS buffer (PBS with 2% FBS and 2 mM EDTA) and then surface stained with antibodies for CD45 (clone 30-F11, Alexa Fluor 700), F4/80 (clone 90, FITC), CD11b (clone M1/70, APC), and CD68 (clone FA-11, FITC) or isotype control for 30 min at 4°C. After washed three times with FACS buffer, cells were stained with DAPI (BD) in FACS buffer to discriminate dead cells and run on a BD FACS Canto II (BD). Data were analyzed with Flow Jo (BD). All antibodies were purchased from Biolegend (USA).

### 2.16. Immunofluorescence

To collect the spinal cords after contusion injury or sham operation mice, mice were anesthetized and then rapidly perfused transcardially with 0.9% saline, followed by 4% PFA. Then, the spinal cords were cryoprotected in 30% sucrose for 3 d at 4°C, before being sectioned. Serial cryostat sections (14 mm thick) were obtained. For immunofluorescent staining, the sectioned slices were permeabilized with 0.3% Triton X-100 in PBS for 30 min and then blocked with 5% BSA for 1 h. After incubation with goat anti-CD31 Alexa Fluor 488-conjugated antibody (1 : 100, R&D, USA), rabbit anti-ki67 (1 : 200, Invitrogen, USA), and rabbit anti-Nrf2 (1 : 200, Proteintech) overnight at 4°C, the sectioned slices were incubated with secondary antibodies anti-rabbit Alexa Fluor 488 or Alexa Fluor 568 (1 : 400, Abcam) for 1.5 h. The slices were mounted with Fluoroshield™ with DAPI (GeneTex Inc.). Signals were analyzed by a fluorescence microscope (Zeiss Apotome 2). Positive cells were quantified using the ImageJ software.

Cell samples were fixed with 4% PFA for 20 min, permeabilized with 0.2% Triton X-100 for 15 min, and blocked with 5% BSA for 30 min and then incubated with primary antibodies overnight at 4°C, followed by secondary antibody incubation for 1.5 h. After washing with PBS, cells were stained with DAPI.

### 2.17. Statistical Analysis

The results were statistically analyzed with SPSS 22.0 (SPSS, Inc.). All data were presented as the means ± standard deviation (SD). Statistical analysis of multiple-group comparison was performed by one-way analysis of variance (ANOVA), followed by the Bonferroni post hoc test. Values of *p* less than 0.05 were considered statistically significant.

## 3. Result

### 3.1. Identification of Microglia and MG-Exos

**Figure 1:**
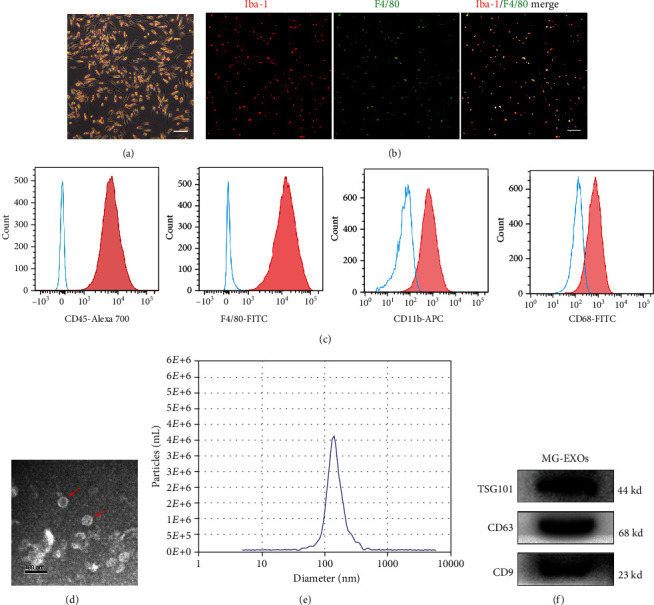
Identification of microglia and MG-Exos. (a) The morphology of microglia. Scale bars = 100 *μ*m. (b) Representative immunofluorescence images of microglia. Iba-1 (red) and F4/80 (green), scale bars = 100 *μ*m. (c) Flow cytometry analysis of the cell markers of microglia. The isotype controls are illustrated as blue dashed curves, and the test samples are illustrated as solid red curves. (d) Representative images of MG-Exo morphology detected by transmission electron microscopy (TEM). Scale bar = 100 nm. Red arrows indicate exosomes. (e) Size distribution assessed by nanoparticle tracking analysis (NTA). (f) Western blotting analysis of specific exosomal surface markers.

Microglia colonies appeared approximately 10-12 days after initial plating. The cells exhibited a typically elongated, either bipolar or unipolar, morphology (Figure 1(a)). We identified the cultured microglia by immunofluorescence costaining of Iba-1 and F4/80 (Figure 1(b)). Flow cytometry analysis revealed that microglia surface markers were highly positive including CD45, F4/80, CD11b, and CD68 (Figure 1(c)). All these features were consistent with the previous studies [35]. TEM and NTA were used to identify particles purified from microglial cells. As shown in Figures 1(d) and 1(e), the nanoparticles exhibited a cup- or sphere-shaped morphology with a size of approximately 80~150 nm. Characteristic biomarkers of exosomes, including TSG101, CD63, and CD9, were also verified by western blotting (Figure 1(f)).

### 3.2. MG-Exos Were Taken Up by Endothelia Cells, Inhibited ROS In Vivo and Vitro

**Figure 2:**
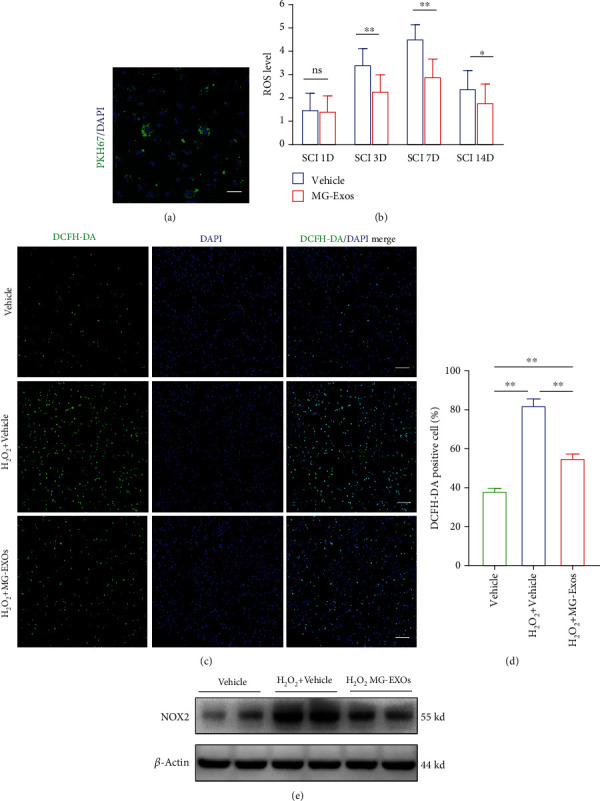
MG-Exos were internalized into endothelia cells and inhibited ROS *in vivo* and *in vitro*. (a) Fluorescence microscopy analysis of PKH67-labeled MG-Exos taken up by bEnd.3. Scale bar = 50 *μ*m. (b) The ROS level of the epicenter of the spinal cord's injury area per group was detected by the DCFH-DA assay throughout the 14-day period post-SCI. (c) Representative fluorescence images of the ROS levels of bEnd.3 treated with H_2_O_2_ plus MG-Exos or Vehicle. DCFH-DA (green) and nuclei (blue); scale bar = 100 *μ*m. (d) Quantitative analysis of the number of DCFH-DA positive cells in (c). (e) Western blotting analysis of the protein levels of NOX2 in bEnd.3 treated with MG-Exos or Vehicle when exposed to H_2_O_2_-induced oxidative stress. The data are presented as the means ± SD; *n* = 6 per group. ^∗^*p* < 0.05 and ^∗∗^*p* < 0.01 compared with different treatment groups.

Oxidative stress is regarded as a critical factor that contributes to the development of a poor microenvironment in SCI repair [4, 36]. To examine the role of MG-Exos played against the toxic effects of oxidative stress, we determined whether MG-Exos could be transferred into endothelia cells firstly. As shown in Figure 2(a), PKH67-labeled MG-Exos (green) were transferred into the perinuclear region of bEnd.3 after 12 h incubation. For functional assays, MG-Exos and an equal volume of Vehicle were injected through the tail vein for 3 consecutive days post-SCI, respectively. And the ROS level of the epicenter (injury site) of the spinal cord was detected by the DCFH-DA assay. The results revealed that the ROS level of the MG-Exos-treated mice was remarkedly decreased in comparison with that in Vehicle-treated mice starting at 3 days post-SCI till 14 days after injury, as shows in Figure 2(b). We next assessed whether the protective effects of MG-Exos could reduce the ROS level in endothelia cells. Fluorescence microscope of DCFH-DA was performed *in vitro*. As shown in Figures 2(c) and 2(d), the level of ROS significantly increased when exposed to the H_2_O_2_ which induced a high level of oxidative stress but decreased after MG-Exo treatment. Further, the protein level of NADPH oxidase 2 (NOX2), a marker of oxidative stress, in both treatment groups (MG-Exos and Vehicle) was analyzed by western blotting (Figure 2(e)). Accordingly, the levels of ROS and the expression of NOX2 detected *in vivo* and i*n vitro* confirm the protective effect of MG-Exos on endothelia cells against oxidative stress.

### 3.3. MG-Exos Promote Survival and Function of Endothelial Cells In Vitro

**Figure 3:**
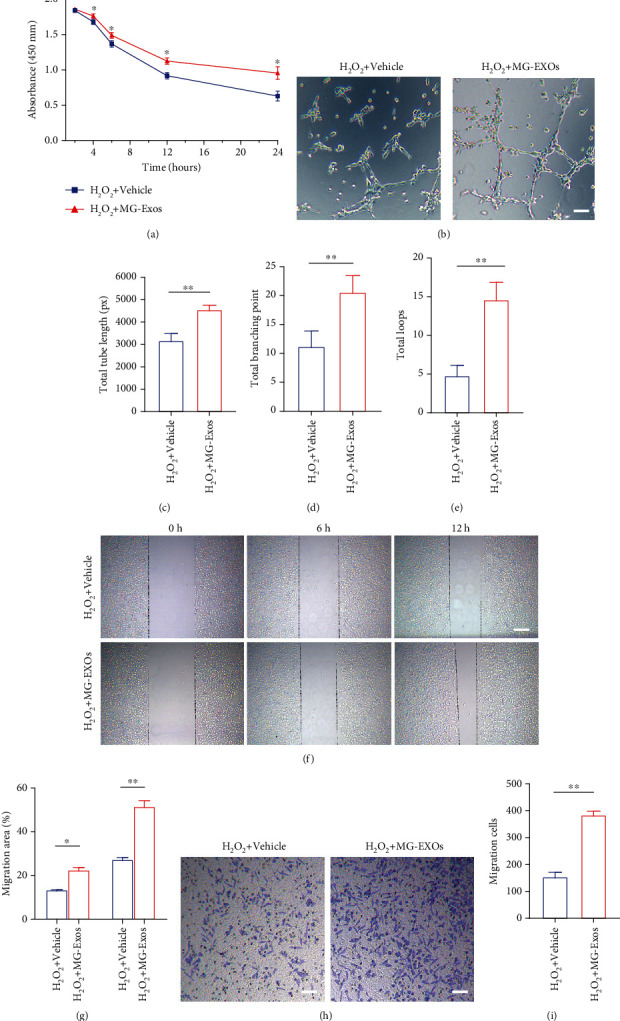
MG-Exos promote survival and function of endothelial cells *in vitro*. (a) CCK-8 analysis of the survival rate of bEnd.3 treated with MG-Exos or Vehicle when exposed to H_2_O_2_-induced oxidative stress. (b) Representative images of bEnd.3 tube formation *in vitro* after H_2_O_2_ plus Vehicle or MG-Exo treatment. Scale bar = 50 *μ*m. (c–e) Quantitative evaluation of the total tube length, total branching points, and total loops in (b). (f) Representative images of bEnd.3 migration in the H_2_O_2_ plus Vehicle or MG-Exo treatment groups in the scratch assay. Scale bar = 250 *μ*m. (g) Quantification of the percentage of migration area in (f). (h) Representative images of transwell experiment in the H_2_O_2_ plus Vehicle or MG-Exo treatment groups. Scale bar = 100 *μ*m. (i) Quantitative evaluation of the number of migration cells in (h). The data are presented as the means ± SD; *n* = 6 per group. ^∗^*p* < 0.05 and ^∗∗^*p* < 0.01 compared with different treatment groups.

To investigate whether the MG-Exos improve endothelial cells' survival and functional recovery when exposed to H_2_O_2_-induced oxidative stress, there were several functional assays performed. CCK-8 assay showed that the survival rate of bEnd.3 was markedly elevated in response to MG-Exo stimulation (Figure 3(a)). The tube formation assay on Matrigel is an *in vitro* model of angiogenesis. As shown in Figure 3(b), bEnd.3 treated with MG-Exos showed a higher number of capillary-like structures compared to the Vehicle-treated group when both groups were exposed to H_2_O_2_. Quantitative measurements revealed that the total tube length, total branching points, and total loops in the MG-Exo-treated group were all drastically higher than that in the Vehicle group (Figures 3(c)–3(e)). MG-Exo treatment also caused a remarkable increase in bEnd.3 migration compared to the control groups under oxidative condition, as evidenced by the scratch wound assay (Figures 3(f) and 3(g)) and transwell migration assay (Figures 3(h) and 3(i)). These findings indicate that MG-Exos elevated the survival rate and promote the functional recovery of endothelial cells under H_2_O_2_-induced oxidative condition.

### 3.4. MG-Exos Promote Vascular Regeneration Post-SCI In Vivo

**Figure 4:**
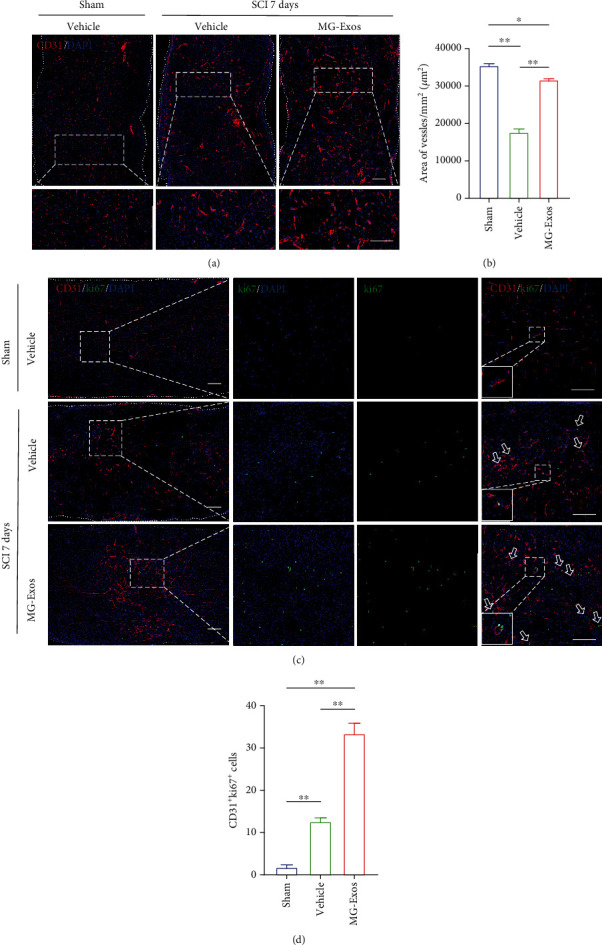
MG-Exos promote vascular regeneration post-SCI *in vivo*. (a) Representative immunofluorescence images of CD31 blood vessels in the epicenter of spinal cord's injury area in each group at 7 days post-SCI. CD31 (green) and nuclei (blue); scale bar = 200 *μ*m and 50 *μ*m (enlarged view). (b) Quantitative evaluation of the area vessels in (a). (c) Representative immunofluorescence images of CD31 (red) and ki67 (green) blood vessels in the mouse's spinal cord in each group at 7 days post-SCI. Scale bars = 200 *μ*m and 50 *μ*m (enlarged view). White arrows indicate CD31^+^ki67^+^ cells. (d) Quantification of the number of CD31 and ki67 double-positive cells in (c). The data are presented as the means ± SD; *n* = 6 per group. ^∗^*p* < 0.05 and ^∗∗^*p* < 0.01 compared with different treatment groups.

We then evaluated the role of MG-Exos played in angiogenesis in a contusive SCI model. Seven days postinjury, a time point at which the proliferative capacity of the blood vessels reached the climax was chosen to evaluate the endothelia cell proliferation [14]. Immunofluorescence analysis revealed that the area of CD31-positive cells in MG-Exo treatment significantly increased in comparison with that in Vehicle-treated mice at 7 days post-SCI (Figures 4(a) and 4(b)). We also performed CD31 and ki67 costaining to test the proliferation of microvascular endothelia cells in the injury site of spinal cord. The result revealed that CD31 and ki67 double-positive cells were rarely observed in the Vehicle-treated mice at day 7 post-SCI, while larger numbers of proliferating microvascular endothelia cells appeared in the injury site when treated with MG-Exos (Figures 4(c) and 4(d)). Taken together, our data suggest that MG-Exos could promote angiogenesis *in vivo*.

### 3.5. MG-Exos Improve Spinal Functional Recovery Post-SCI

**Figure 5:**
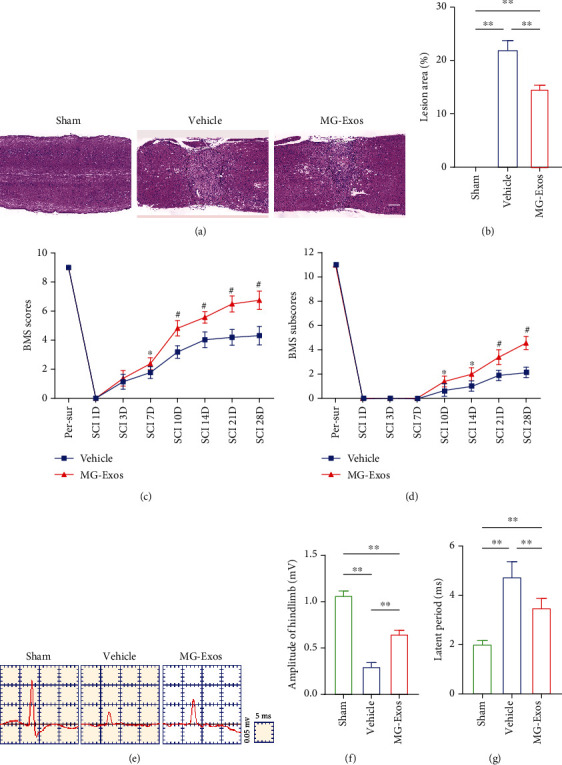
MG-Exos improve spinal functional recovery after SCI. (a) Representative H&E staining of the longitudinal epicenter injury of spinal cord in each group at 28 days post-SCI. Scale bar = 1 mm. (b) Quantification of the lesion area in (a). (c) Distribution of the BMS scores per group after SCI throughout the 28-day period. (d) Distribution of the BMS subscores per group at 0, 1, 3, 7, 14, 21, and 28 days post-SCI. (e) Representative electrophysiological traces in each group at 28 days post-SCI. (f, g) Quantification of the amplitude and latent period of MEPs in (e). The data are presented as the means ± SD; *n* = 6 per group. ^∗^*p* < 0.05 and ^∗∗^*p* < 0.01 compared with different treatment groups.

To further determine the therapeutic effects of MG-Exos on neurological functional recovery after SCI, histological analysis and behavior tests were performed. H&E staining was carried out to explore the extent of spinal cord repair. As shown in Figures 5(a) and 5(b), the injury area remarkedly decreased in the MG-Exo-treated mice compared to the Vehicle-treated mice at 28 days post-SCI. In consistent with H&E, during the motor function test, the mice treated with MG-Exos showed drastically elevated locomotor recovery after SCI, as indicated by increased BMS scores and BMS subscores starting at 7 days post-SCI till 28 days after injury (Figures 5(c) and 5(d)). In addition, the electrophysiological analysis of motor-evoked potentials (MEPs) indicated the amplitude of the MG-Exo-treated mice significantly increased compared to the Vehicle-treated mice at 28 days post-SCI (Figures 5(e) and 5(f)); moreover, the latent period was shortened in the MG-Exo-treated mice (Figure 5(g)). These results demonstrated MG-Exos had the protective effects on endothelia cells against functional loss in SCI.

### 3.6. MG-Exos Minimized the Negative Impacts of Oxidative Stress in Endothelia Cells by Modulating the keap1/Nrf2/HO-1 Signaling Pathway

**Figure 6:**
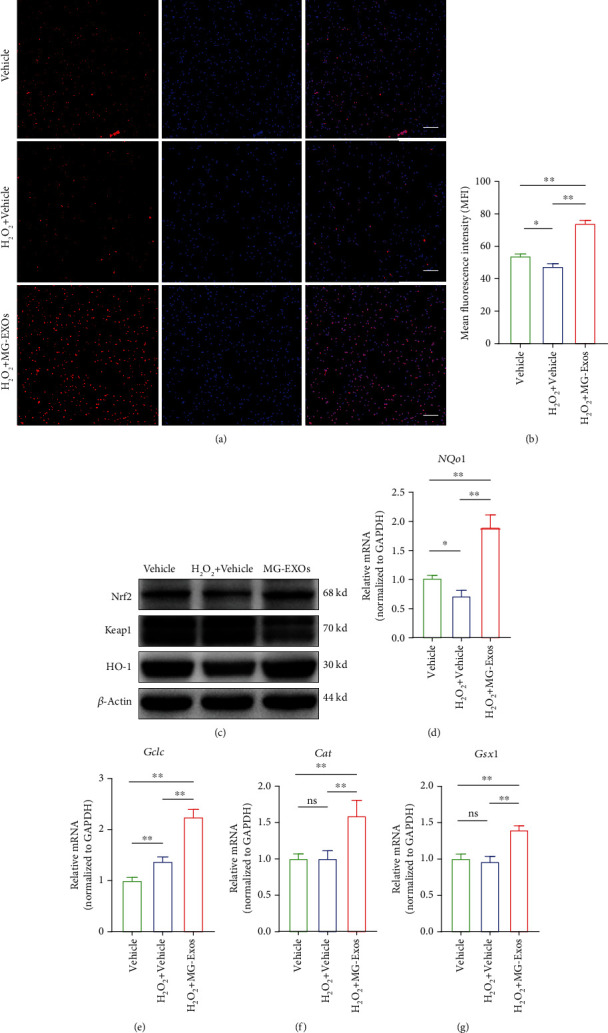
MG-Exos protect endothelia cells against the oxidative effects by modulating the keap1/Nrf2/HO-1 signaling pathway. (a) Representative immunofluorescence images of the Nrf2's expression of bEnd.3 after Vehicle or MG-Exo treatment when exposed to H_2_O_2_-induced oxidative stress. Scale bar = 100 *μ*m. (b) Quantification of mean fluorescence intensity (MFI) in (a). (c) Western blotting analysis of the protein levels of keap1, Nrf2, and HO-1 in H_2_O_2_ plus Vehicle or MG-Exo treatment groups. (d–g) qPCR verification of the mRNA levels of downstream antioxidative-related genes including *NQo1*, *Gclc*, *Cat*, and *Gsx1* per group when exposed to H_2_O_2_-induced oxidative stress. The data are presented as the means ± SD; *n* = 6 per group. ^∗^*p* < 0.05 and ^∗∗^*p* < 0.01 compared with different treatment groups.

To explore whether the keap1/Nrf2 signaling pathway involved in the endothelia cells' protective process against oxidative stress after MG-Exo treatment, levels of keap1, Nrf2, and HO-1 proteins were measured using immunofluorescence staining and western blotting. As shown in Figures 6(a) and 6(b), immunofluorescence staining indicated that excessive oxidative stress would decrease the expression levels of Nrf2, while MG-Exo treatment reversed the change. In accordance with the immunofluorescence results, the western blotting showed that the protein levels of keap1 in the MG-Exo-treated group after exposed to H_2_O_2_ were significantly downregulated compared to the Vehicle group; however, the protein levels of Nrf2 and HO-1 were upregulated in the MG-Exo-treated group (Figure 6(c)). qRT-PCR was further carried out in H_2_O_2_-treated bEnd.3 to validate the mRNA of downstream antioxidative-related genes, which were reported to be closely (*NQo1*, *Gclc*, *Cat*, and *Gsx1*) associated with the keap1/Nrf2 pathway [37–39]. As quantified analysis indicated, the mRNA levels of *NQo1*, *Gclc*, *Cat*, and *Gsx1* were significantly higher in the MG-Exo-treated group (Figures 6(d)–6(g)). These results suggest that MG-Exos inhibit oxidative stress in endothelia cells via modulating the keap1/Nrf2/HO-1 signaling pathway.

## 4. Discussion

SCI is a worldwide devastating disease of the central nervous system companied by a long-term disability and high mortality [1, 40]. Until now, numerous treatment strategies have been used to ameliorate the neurological regeneration of the spinal cord after SCI, but unfortunately, none have been convincingly effective in improving the neurological function of SCI patients. In our present study, we found that microglia-derived exosomes protected blood vessel against the toxic effects of high oxidative stress and improved spinal cord functional recovery. Our results revealed that MG-Exos could reduce the level of H_2_O_2_-induced ROS of endothelia cells *in vivo* and the ROS level in the epicentral spinal cord in the mice's SCI model. We further confirmed that MG-Exos could promote endothelia cells' survival and function *in vitro* and new vessel formation *in vivo*. The activation of the keap1/Nrf2/HO-1 signaling pathway was the potential mechanisms underlying the MG-Exo treatment for SCI.

Emerging evidences suggested that microglia could influence the formation of new blood vessels and vascular development in the central nervous system and retina [25, 26]. In spinal cord tissue, microglia and microvascular endothelial cells have a compact anatomical structure. During SCI, microglia would form clusters around the vasculature, and then, phagocytose damaged vascular tissue [23]. Subsequently, they would secrete multiple factors such as VEGF, basigin-2, and FGF2, which are known to promote angiogenesis and tissue repair [23, 27]. Meanwhile, microglia-lacking mice, with which some other experiments conducted, were found to have reduced developing retinal vasculature [28]. Theses evidences suggested that microglia could promote the reconstruction of blood vessel following central nervous system injury. Moreover, numerous studies have shown that mesenchymal stem cells (MSCs) were mainly relied on their paracrine function rather than their capacity for differentiation to repair tissue damage [41, 42]. This means that exosomes could perform most of the functions of their parental cells. Therefore, we choose microglia exosomes to perform our study.

As we know, neurovascular communication is essential for the homeostasis and well-functioning of the neuronal compartments in the central nervous system [43]. The researches of neurovascular opened a new perspective for current studies of repairing the damaged central nervous system. The reconstruction of damaged neural networks after SCI needed microvasculature to provide nutrients and maintain their homeostasis [14]. Furthermore, the newborn microvasculature could provide the scaffolds for regenerating axons which might grow along the new vessels; otherwise, the abnormalities in angiogenesis would delay neural tissue regeneration [12]. Yuan et al. have reported that pericyte-derived exosomes could improve the endothelial ability of regulating blood flow and then could promote the recovering degree of neurological function after SCI [44]. As shown in our previous studies, the exosomes derived from human urine stem cells could enhance spinal cord functional recovery via promoting vascular regeneration post-SCI [45]. Thus, promoting angiogenesis and restoring blood flow to the injured cord may provide an essential foundation for spinal cord repair and recovery. In our current study, we observed that MG-Exos could drastically increase the number of newly formed blood vessels and could significantly improve the endothelial cells' proliferative ability in the epicentral injury of spinal cord. Our neurological function assays also revealed that MG-Exos could promote the recovery of neurological function post-SCI. These results suggest that the profunction recovering action of MG-Exos is likely attributable to their stimulatory effects on endothelial angiogenesis.

On the other hand, the oxidative stress along with the generating of free radicals drastically leveled up following SCI. They were the main pathological factors of secondary damage, and they also could lead to endothelial dysfunction and the limited endogenous repair of microvasculature during SCI [10]. ROS, a main form of oxidants, could cause lipid peroxidation, protein inactivation, DNA fragmentation, and ultimately cells' dysfunction or even death. Consistent with results of previous researches [22], the increase in levels of oxidative stress products (ROS) and NOX2, a marker of oxidative stress, was found in both the H_2_O_2_-induced bEnd.3 cells and the SCI model. Treatment with MG-Exos was found to significantly decrease the ROS production and the expression level of NOX2 in the H_2_O_2_-induced bEnd.3 cells as well as the SCI model. *In vitro*, functional assays of endothelia cells revealed that MG-Exos markedly improved the survival and function of endothelial cells when being exposed to H_2_O_2_-induced oxidative stress. These findings indicate that MG-Exos have the ability to resist oxidative stress.

To investigate the detailed molecular mechanism of antioxidation, the keap1/Nrf2/HO-1 signaling pathway was evaluated. The activity of Nrf2 was crucial to regulate intracellular oxidative stress status [17, 46]. In normal physiological conditions, Nrf2 anchored in the cytoplasm by keap1 which could promote the ubiquitination of Nrf2. Then, the ubiquitinated Nrf2 would be rapidly degraded by proteasome [47]. When cells were exposed to ROS or free radicals, Nrf2 dissociated from keap1 and quickly transferred into the nucleus and then binded to the antioxidant response element (ARE), exerting antioxidation through promoting the transcription of its downstream antioxidative-related genes, such as Heme Oxygenase-1 (HO-1), NAD (P) H quinone oxidoreductase 1 (NQo1), Glutamate-cysteine Ligase Catalytic subunit (Gclc), Catalase (Cat), and GS Homeobox 1 (Gsx1) [17]. In our present study, we observed that the protein levels of Nrf2 and HO-1 were markedly decrease in H_2_O_2_-induced bEnd.3 cells, but keap1's expression had no change. These findings were consistent with previous research results. However, treatment with MG-Exos significantly increased the levels of Nrf2 and HO-1 proteins. Also, the expression of keap1 was remarkedly downregulated in H_2_O_2_-induced bEnd.3 cells. We further validated that the expression of downstream antioxidative-related factors of keap1/Nrf2/HO-1, including *NQo1*, *Gclc*, *Cat*, and *Gsx1*, had varying degrees of elevation *in vitro*. These results indicated that MG-Exos could exert an antioxidant function through the keap1/Nrf2/HO-1 signaling pathway.

## 5. Conclusions

In conclusion, our results confirmed that MG-Exos could exert their influences as antioxidants against oxidative stress via activating the keap1/Nrf2/HO-1 pathway. Moreover, MG-Exos promoted the survival as well as functions of endothelia cells and the recovery degrees of neurological function *in vitro* and *vivo*. Therefore, MG-Exos may be able to protect endothelia cells from oxidative stress and promote their restoration, thus enhance the spinal cord functional recovery during SCI treatment.

## Figures and Tables

**Table 1:** All primer sequences used for qRT-PCR.

*NQo1*	Forward primer	ATGGGAGGTGGTCGAATCTGA
	Reverse primer	GCCTTCCTTATACGCCAGAGATG
*Gclc*	Forward primer	GGGGTGACGAGGTGGAGTA
	Reverse primer	GTTGGGGTTTGTCCTCTCCC

*Cat*	Forward primer	AGCGACCAGATGAAGCAGTG
	Reverse primer	TCCGCTCTCTGTCAAAGTGTG

*Gsx1*	Forward primer	CTTCCCTCCCTTCGGATCG
	Reverse primer	GTCCACAGAGATGCAGTGAAA

## Data Availability

The data used to support the findings of this study are included in the article. The authors stated that the data underlying the findings of this manuscript is available to share.
